# The impact of architectural modifications on relative resistance to fluid flow in ventricular catheters

**DOI:** 10.3389/fbioe.2024.1519499

**Published:** 2025-01-29

**Authors:** Rajesh Kumar Madhavan, Ahmad Faryami, Nathan Tappen, Pranav Gopalakrishnan, Shaheer H. Ajaz, Christopher Roberts, Carolyn Harris

**Affiliations:** 1 Department of Biological Sciences, Wayne State University, Detroit, MI, United States; 2 Department of Biomedical Engineering, Wayne State University, Detroit, MI, United States; 3 Department of Chemical Engineering, Wayne State University, Detroit, MI, United States; 4 School of Medicine, Wayne State University, Detroit, MI, United States

**Keywords:** ventricular catheter (VC), catheter resistance, bench top model, catheter architecture and design, hydrocephalus

## Abstract

**Introduction:**

Although many ventricular catheter designs exist for hydrocephalus treatment, few standardized studies assess outflow resistance and the impact of design modifications on shunt drainage. This study represents the *in-vitro* assessment of various architectural modifications on catheter flow rate and pressure, focusing on bulk outflow dynamics and occlusion with whole blood-inoculated cerebrospinal fluid.

**Methods:**

Catheters were manufactured utilizing a novel catheter production setup with 16 variations from standard catheters, including but not limited to changes in: hole number, hole dimensions, catheter lumen dimension, and catheter lumen impingement. These catheters were tested in a portable custom-made ventricular catheter testing device to analyze relative resistance to flow between catheter designs. A subset of catheters with varying lumen diameters was tested in 0.30 mL/min saline flow with 2.5% blood to simulate early blood exposure.

**Results:**

With increasing hole and lumen diameter, we found a significant decrease in overall catheter relative resistance using DIH_2_0 (*P* < 0.001 and P < 0.002 respectively, n = 5). With increasing lumen diameters, blood assays showed a significant increase in the time to complete obstruction (*P* = 0.027, n = 5). Lumen impingement, representing one obstruction-based pinch point in the lumen, showed a considerable increase in relative resistance as obstruction diameter increased and lumen diameter at the pinch point decreased (*P* = 0.001, n = 5). Removal of specific catheter hole rows trended toward an increase relative resistance after 75% of catheter holes were blocked, but the effect in relative outflow resistance is otherwise minimal (*P* > 0.05, n = 5) and no effect was observed with blocking segments.

**Conclusion:**

This study implemented a novel method of rapid catheter manufacturing to systematically produce ventricular catheters with specific catheter architecture. By testing variables independently, we found that catheters with changes to the lumen diameter had the most dramatic shifts in overall relative resistance between catheter designs. Similarly, testing in the acute *in-vitro* blood assay demonstrated that smaller diameter catheters have a higher propensity to obstruct with blood compared to catheters with larger diameter. Relative resistance impacts fluid outflow efficiency, which may translate to clinical outcomes for hydrocephalus patients. These findings help us understand catheter architectural effects on resistance and inform future designs for specific ventricle morphologies.

## Introduction

Hydrocephalus is a multifaceted and complex neurological disease characterized by an abnormal accumulation of cerebrospinal fluid (CSF) within the cerebral ventricles (Lee et al., 2022; Isaacs et al., 2018; Rekate, 2009; Brinker et al., 2014; Greitz et al., 1992; Milhorat, 1975; Penn et al., 2011). The cerebral ventricles typically enlarge, compressing the brain and elevating intracranial pressure (ICP), which can then lead to other physiological concerns (Stone et al., 2013). While the predominant approach to managing hydrocephalus involves the surgical implantation of shunt systems to redirect excess CSF away from the brain, this method is plagued by considerable failure rates, with approximately 50% of shunts failing within 2 years and up to 85% requiring shunt revisions within 10 years post-implantation (Lee et al., 2022; Kulkarni et al., 2013; Hanak et al., 2017; Drake et al., 2000; Kestle et al., 2001; Borgbjerg et al., 1995; Malm et al., 2004; Cheatle et al., 2012; Gopalakrishnan et al., 2023). Ventricular catheter obstruction stands out as a primary cause of shunt failure, accounting for approximately half of all pediatric catheter failures (Hanak et al., 2016; Hariharan et al., 2021). While the exact mechanisms of shunt obstruction are still under investigation by us and others, it is hypothesized that the obstruction of ventricular catheters arises from a complex interplay of physical shunt factors and its subsequent biological response. These include elements such as contact- or flow-mediated infiltration of choroid plexus, contact- and/or growth-mediated parenchymal tissue invagination, contact- and/or growth-mediated ventricular wall pull in, blood-dependent sequelae, and the accumulation of inflammatory cells (Hanak et al., 2016; Harris and McAllister, 2012; Takahashi et al., 1998; Kraemer et al., 2018; Elkaim et al., 2019). The catheter’s physical geometric considerations include catheter hole geometry, hole placement, and diameter assuming catheter insertion with adoption of existing well-accepted insertion protocols (Bigner et al., 1985; Del Bigio and Bruni, 1986; Schoener et al., 1991; Sekhar et al., 1982; Thomale et al., 2010; Galarza et al., 2018).

Efforts to mitigate ventricular catheter obstruction have included altering external catheter traits to modify cell adhesion properties and reduce immune response within the catheter, aiming to prevent blockages (Hanak et al., 2018). The impact of the ventricular catheter architecture and its relationship to CSF dynamics is under investigation by us and others (Lin et al., 2003). Still, it remains unclear how these factors independently influence biological response. Previous studies have indicated that catheter architectural characteristics significantly affect macrophage and astrocyte adhesion *in-vitro*, emphasizing the connection between biological-related blockages and architectural geometry (Galarza et al., 2018; Harris and McAllister, 2011). Other studies have analyzed shear forces to understand how catheter architecture affects CSF flow through the holes, but these investigations have largely been limited to computer simulations and existing commercially available catheter variants that differ in multiple architectural dimensions (Penn et al., 2011; Galarza et al., 2018; Ginsberg et al., 2000; Lee et al., 2020).

Commercially available catheters vary across several independent dimensions: hole diameter, spacing between holes, lumen diameter, tip geometry, biomaterial, number of segments (regions along the catheter relevant to drainage), and number of rows. This diversity has contributed to the difficulty of retrospective analysis of explanted catheters and systematic analysis of the impact these elements have on shunt obstruction. Currently, there is no cost-effective method of producing batches of silicone ventricular catheters with custom elements to systematically investigate the impact of architectural changes in ventricular catheters. The result is a fragmented understanding of how catheter architecture influences flow resistance, and by extension, patient health. This underscores a pressing need for a systematic approach to catheter design research.

Numerous studies have demonstrated that ventricular catheter performance is strongly influenced by its architecture, including lumen diameter, hole size, and arrangement. For example, previous research has shown that catheter hole size and number significantly affect resistance and flow efficiency (Thomale et al., 2010; Ginsberg et al., 2000). Other studies have emphasized that wall shear stress plays a critical role in reducing astrocyte obstruction in ventricular catheters, highlighting the importance of fluid dynamics in catheter design (Lee et al., 2020). Additionally, investigations into catheter geometry have revealed that design features can influence blood-induced obstructions, such as clotting and flow resistance (Woo et al., 2019; Vandersteene et al., 2019). These findings underscore the necessity of systematically evaluating catheter architecture, which is the main goal of this study.

To better understand how different catheter architectural dimensions affect outflow resistance to CSF flow, we standardized the fabrication of small-batch catheters with varying hole diameter, lumen diameter, number of holes, and hole placement. We then also added consistent artificial obstructive masses to mimic impingement to the catheter lumen following tissue obstruction—ranging from row and segment obstructions to artificial lumen obstructions. Reliable production of catheters combined with an accurate mode of measuring relative resistance changes between catheters can provide concrete evidence of how distinct alterations of catheter fundamental dimensions impact innate resistance. Precise measurements of outflow resistance from outside the catheter to the catheter lumen provide concrete evidence of how specific catheter dimensions affect flow resistance. This understanding is crucial for ventricular catheter optimization for flow, pressure, and biologic response.

## Materials and methods

### Catheter design and production system

**FIGURE 1:**
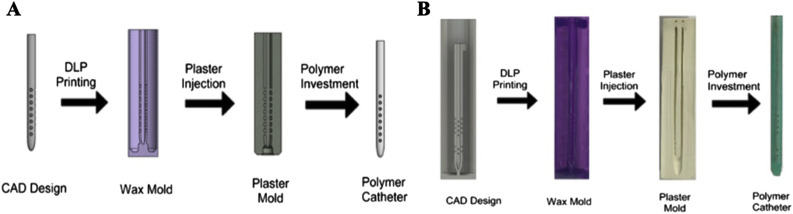
Illustration and real Catheter Research and Fabrication Technology System (CRAFTS). **(A)** An illustration of the CRAFTS from Computer-aided Design (CAD) to Digital Light Processing (DLP) printing of wax mold (purple). After plaster injection plaster mold is set for polymer injection into an open cavity. **(B)** The real production process.

**FIGURE 2:**
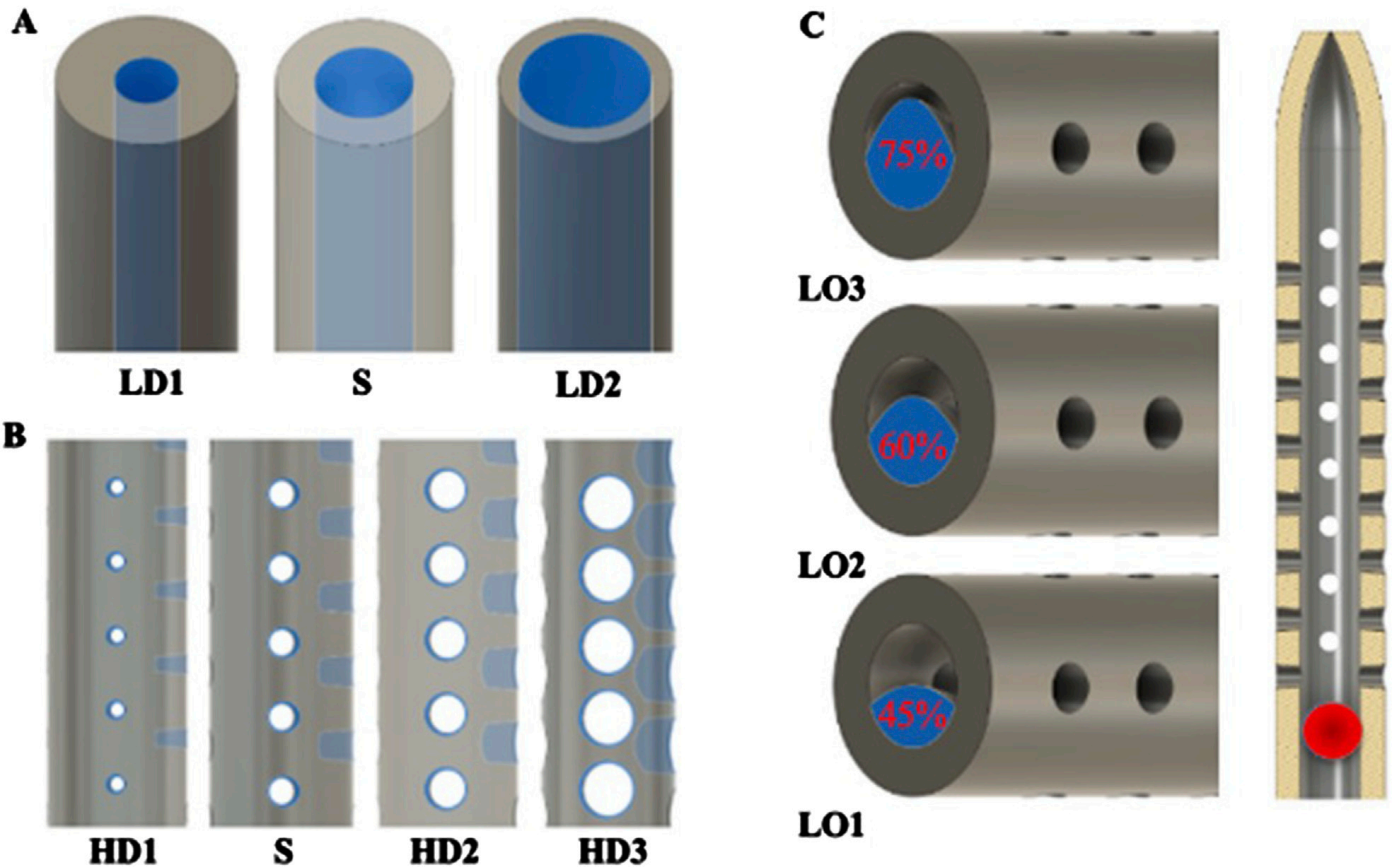
Computer-Aided Design (CAD) manipulation of catheter architectural dimensions. **(A)** CAD modification of the lumen diameter of standard (S) to produce smaller and larger lumen. The blue highlighted region is press-pulled evenly in or out to make the inner lumen diameter smaller or bigger, respectively. **(B)** CAD modification of the hole diameter of standard (S) to produce smaller or bigger holes. Highlighted blue areas were press-pulled evenly in or out to make the holes smaller or larger in diameter, respectively. **(C)** Depiction of how artificial luminal obstructions were produced inside the lumen. Highlighted areas in blue represents the cross-sectional area of the maximal obstruction. Highlighted circle in the red shows the location of the ball-point obstruction along the catheter.

Catheter design and production was done using our novel Catheter Research and Fabrication Technology System (CRAFTS) (Figure 1). A three-dimensional (3D) catheter model was rendered using a Resonance-scanning confocal microscopy (RS-G4 uprightmicroscope, Caliber ID, Andover, MA, United States). The scanned catheter was converted to an f3d file, so it could be imported to Fusion 360 (Fusion 360, Inc., San Francisco, CA, United States). Once the standard catheter was imported, the catheter’s architectural dimensions mentioned prior were systematically altered. Modifications to the hole diameters were performed using the press-pull function within Fusion 360, allowing for precise adjustments to the hole diameter by manipulating the digital geometry while maintaining the conical shape and angle. Figures 2A, B illustrates the holes and lumen size of the standard being manipulated within the software. Once alterations were completed, each file was converted into Standard Tessellation Language (STL) files, which were then processed into Partial Range Zone (PRZ) files on Lychee Slicer (Mango 3D, Inc., Mérignac, Nouvelle-Aquitaine, France). Individual files were printed in separate batches using a Phrozen 8k SLA printer with a wax-based resin (Phrozen Tech CO., LTD., Hsinchu City, Taiwan).

**FIGURE 3:**
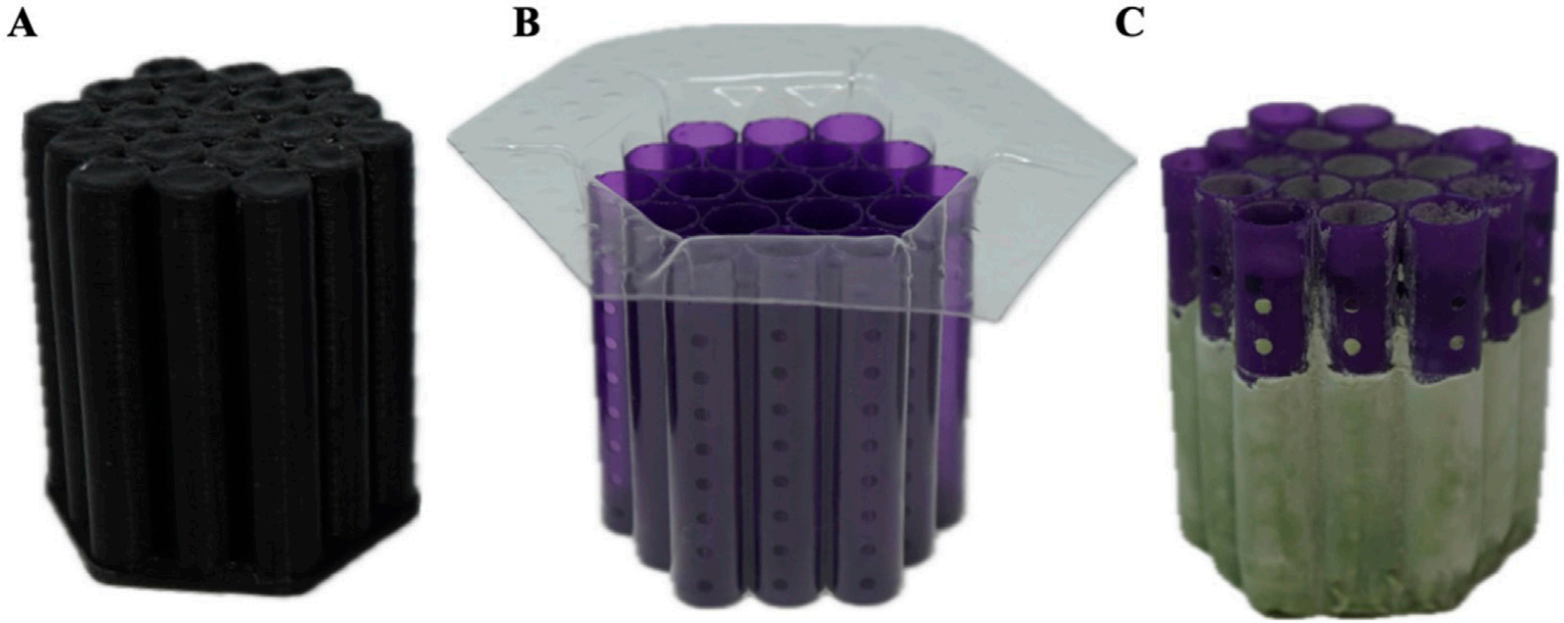
Custom Polyethylene Terephthalate Glycol (PETG) Insert. **(A)** The insert is fabricated using vacuum suction forming technology with a mold designed to replicate the catheter structure. **(B)** The funnel is engineered to tightly compress the wax catheter molds, while the extended height of the insert acts as a funnel. **(C)** The funnel directs the flow of plaster into the mold cavity, ensuring complete and uniform filling.

Once printed, the parts were cleaned and cured using common SLA procedures. After, the catheters were added to a custom insert with a built-in funnel (Figure 3). A batch of plaster was made using plaster powder and distilled water, according to the manufacturer’s recommendations. The distilled water and plaster were mixed in a standardized procedure involving a ZKJ-3 Mixing Vibrating Machine, which was set to mix for 3 min at 11.6 psi, as per the manufacturer’s guidelines (PuTian City OuBo E-Business Co., Ltd., Putian Chengxiang District, Fuzhou, China) (Olena, 2019; Zhong-xu, 2005). The plaster was poured into the insert containing the resin catheter shells while set on a common vibrating plate, which ensured the plaster filled in the complex details of the catheter. Once this was visually seen, the plaster-filled inserts were put inside a Thermo Scientific Sorvall Legend XTR Challenge centrifuge and spun at 500 rpm for 3 min (Thermo Fisher Scientific, Inc., Waltham, MA, United States). The extracted molds were set to cure overnight in a dehydrator that removed excess moisture.

Next, the insert was removed to expose individual plaster-filled resin molds that were then put inside a benchtop kiln. The kiln was programmed according to the manufacturer’s guidelines for the resin, which was burnt off, leaving an empty cavity of the catheter within the plaster shells. These plaster shells were placed in the same insert, but this time for material extraction. Commercial catheters vary from 40 to 60 durometer in hardness of material; here, we used 50D Ecoflex Platinum Cure Silicone Rubber (Smooth-On, Inc., Macungie, PA, United States). Parts A and B of silicone were mixed in a 1:1 ratio, and five drops of silicone rubber pigment were added (DecorRom, Shenzhenshi Baishifuyou Trading Co., Ltd., Longgang, Shenzhen). The color coding identified each catheter variation. The parts were physically agitated then centrifuged at 3,200 rpm to mix and remove air bubbles for 3 min.

The silicone was poured inside the insert until all the empty cavities in the plaster shells were full. Using a common benchtop vacuum chamber, the air bubbles were further removed. Once the silicone was set, the plaster shells were dissolved using a generic plaster solvent. The catheters were further cleaned using a DK sonic ultrasonic cleaner at full waves for 90 min (Shenzhen Dekang Cleaning Electronic Appliance Co., Ltd., Shenzhen, Guangdong, China). Seventeen catheter variations, including the standard model, were produced, cut to 30 mm in length, and labeled by name and sample number, with each type having a sample size of five (n = 5).

### Experimental investigations

Three hypotheses of ventricular catheter architecture were evaluated: (1) relative resistance of holes in row and segment obstruction groups will not be significantly different from standard catheter, (2) relative resistance will decrease when hole diameter or lumen diameter is increased, (3) lumen obstructions significantly increase the resistance of a catheter.

#### Row and segment occlusions

The row obstruction (RO) group used a standard catheter with four rows, which was further categorized into three variations: 1RO (three rows open), 2RO (two rows open), and 3RO (one row open). This was done to assess whether decreasing the number of open rows from four to one significantly impacts the shear resistance of a catheter, a comparative analysis was performed. Complete obstruction (CO) catheters were also made (n = 3) to depict cases where all rows were completely obstructed. Segment (Sg.) obstruction has four variations with each Sg. obstructed individually. Sg.1, Sg.2, Sg.3, and Sg.4 are illustrated in Table 1. The goal of this group is to determine if holes obstructed in different segments affect the catheter’s resistance, and to identify whether there is any statistical significance in resistance between holes obstructed closer to the tip *versus* those further toward the distal end.

**TABLE 1:** Overview of Catheter Variations Manufactured in This Study. This table outlines all experimental and control groups included in the study, along with their corresponding group identifiers, modifications applied to the samples in each group, and detailed descriptions of the experimental groups. For the lumen obstruction group, the implantation sites of obstructions are marked with a red circle. All catheter descriptions are provided in relation to their orientation from the tip to the tail.

Group name	Description of each group	Variations	Identifier	Images
Standard (S)	Four rows with 8 holes each, based on a commercial catheter.	Manufactured Standard	S	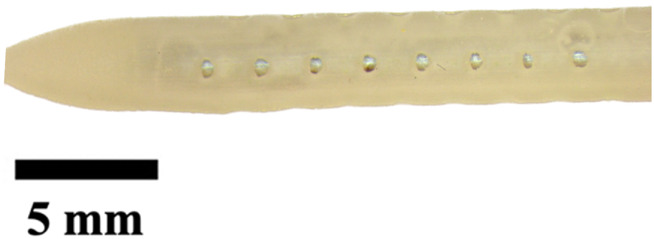
Row Obstruction (RO)	Increasing (continuous) obstruction of all holes within each row. Starting from one row (tip to tail holes) obstructed to the most extreme where 3 out 4 rows have all obstructed holes.	1 row obstructed	1RO	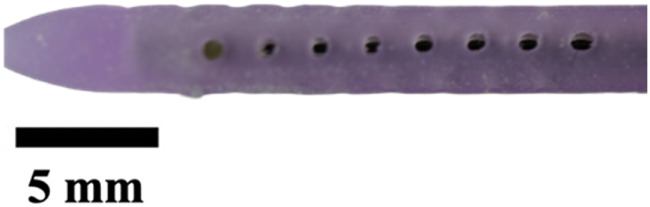
		2 rows obstructed	2RO	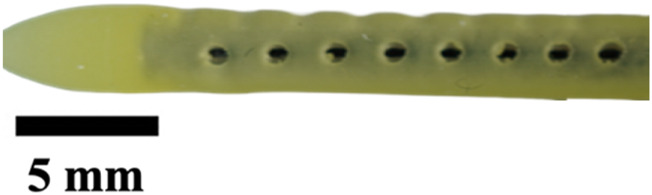
		3 rows obstructed	3RO	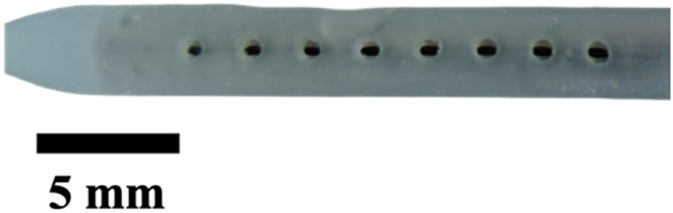
Complete Obstruction (CO)	All holes in each row/column are obstructed.	4 rows obstructed completely	CO	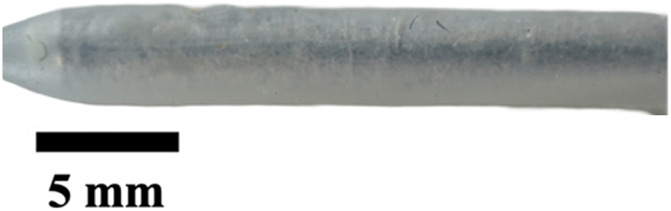
Segment Obstruction (Sg.)	Variable obstruction of different columns of the catheter. There are 4 columns; each contains 2 holes on each of the 4 rows. Only one column is obstructed at a time.	1st column obstructed	Sg.1	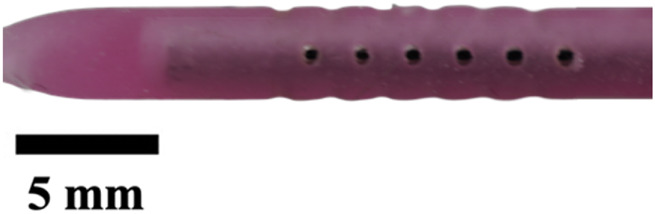
		2nd column obstructed	Sg. 2	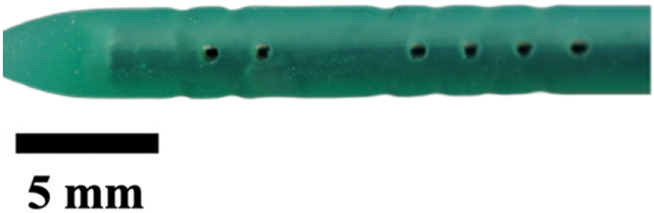
		3rd column obstructed	Sg. 3	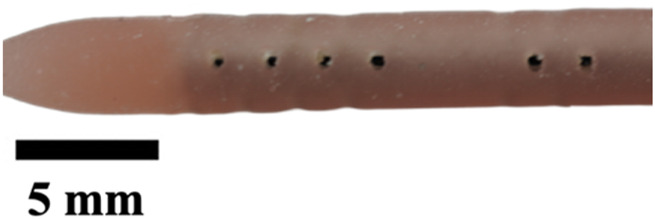
		4th column obstructed	Sg. 4	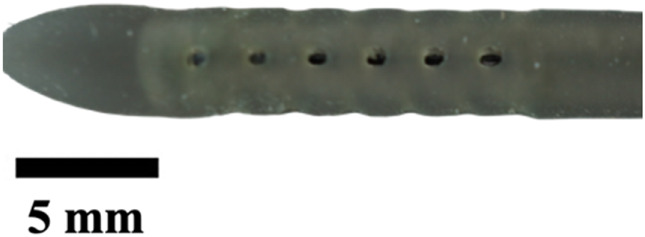
Hole diameter (HD)	All holes of catheters are either increased (+) or decreased (−) in comparison to standard negative control catheters.	−0.10 mm from standard	HD1	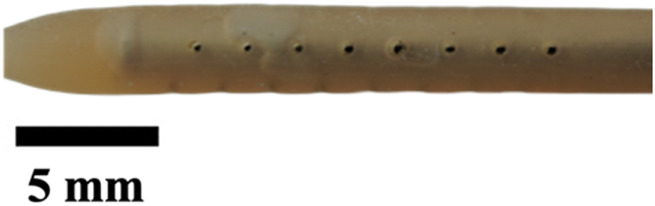
		+0.10 mm from standard	HD2	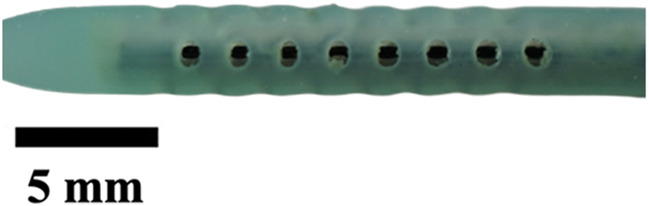
		+0.25 mm from standard	HD3	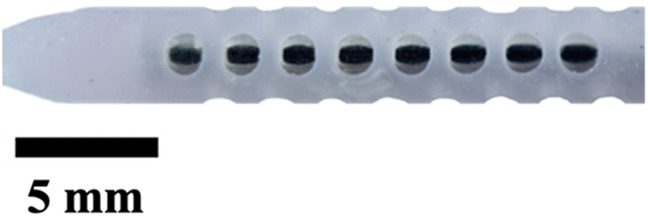
Lumen Diameter (LD)	Lumen thickness is either increased (+) or decreased (−) in comparison to the standard negative control catheters	−0.25 mm from standard	LD1	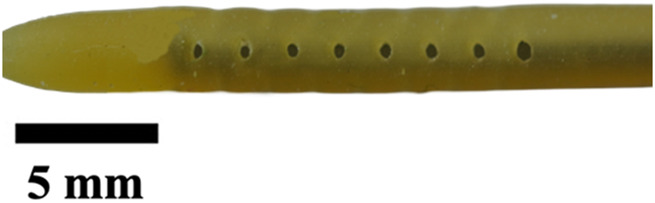
		+0.25 mm from standard	LD2	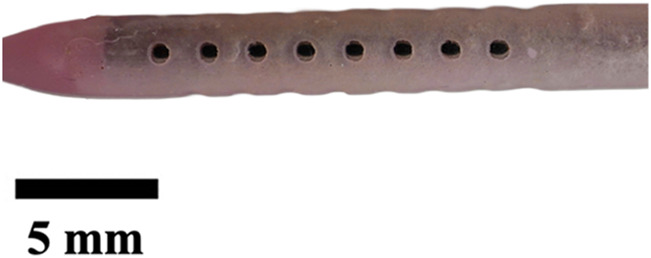
Lumen Obstruction (LO)	Different diameters of sphere-like obstructions are projected from one side of the inner lumen. The obstruction is located right under the last column of the catheter.	Diameter: 0.68 mm Percent Occluded: 45%	LO1	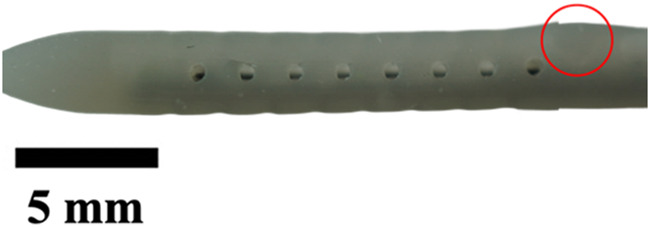
		Diameter: 0.98 mm Percent Occluded: 60%	LO2	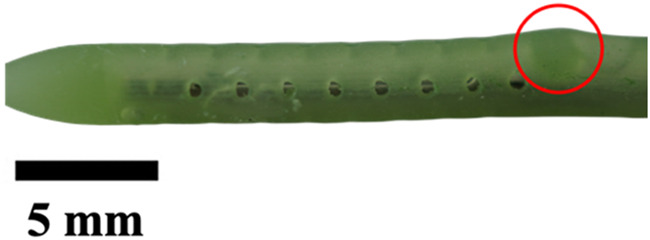
		Diameter: 1.18 mm Percent Occluded: 75%	LO3	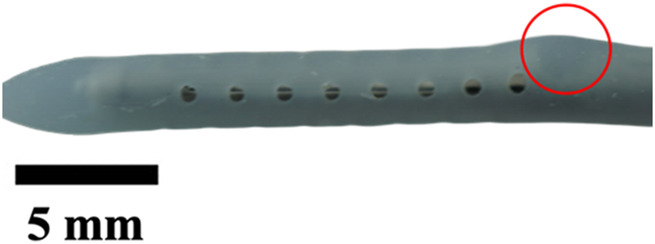

#### Hole diameter and lumen diameter

**FIGURE 4:**
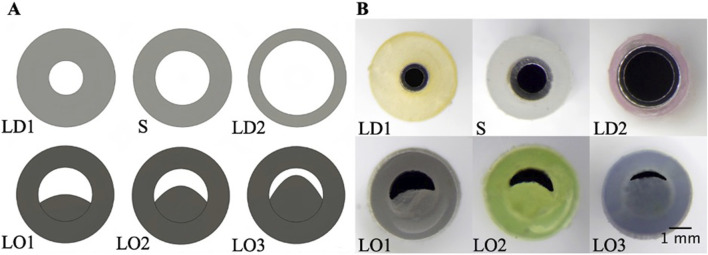
Cross-sectional view of CAD and real manufactured catheters. **(A)** shows CAD cross-sectional view of inner lumen variations (LD1, S, and LD2) and inner lumen obstruction (LO1, LO2, LO3). **(B)** shows an actual cross-sectional view of inner lumen variations (LD1, S, and LD2) and inner lumen obstruction (LO1, LO2, LO3).

The hole diameter group has 3 variations from the standard catheter, these include HD1 (0.3482 mm), S (0.5475 mm), HD2 (0.7458 mm), and HD3 (1.0421 mm). The inner lumen diameter group has 2 variations from the standard 1.36   mm, where LD1 is 0.86mm, and LD2 is 1.86 mm (Figure 4). Architectural dimensions of diameter for both the holes and the inner lumen were manipulated to discern how much the resistance of a catheter is affected by these variables. It will be interesting to denote which plays a more significant role in the resistance of a catheter.

#### Spherical artifact inside the lumen of the catheter

Beyond the architectural modifications of a standard catheter, this study introduces three catheter groups differentiated by the size of an artificially created spherical lumen obstruction. These obstructions are designed to evaluate how variations in inner lumen occlusions (LO) impact resistance. The variations include LO1, LO2, and LO3 which correspond to increasing levels of maximal cross-sectional occlusion, measured at 45%, 60%, and 75%, respectively (Figure 4). These progressively greater degrees of occlusion provide a basis for analyzing their impact. Figure 2C offers a detailed visualization of the artifact creation process, emphasizing the methodology and the distinctions among the three occlusion levels.

### Visual inspection and imaging

All manufactured catheters were visually inspected after post-processing to ensure structural integrity and dimensional accuracy. Imaging was conducted using a Trinocular Stereo Zoom Microscope (SM-4TZ-144A, AmScope, United States) equipped with a 0.5x Barlow lens, and a Sony Alpha ZV-E10 APS-C Interchangeable Lens Mirrorless Camera (Model: ZV-E10, Sony Corporation, Japan) with a 24.2 MP sensor. This setup provided high-resolution imaging and detailed visualization of catheter features and potential irregularities.

### Method of data collection

**FIGURE 5:**
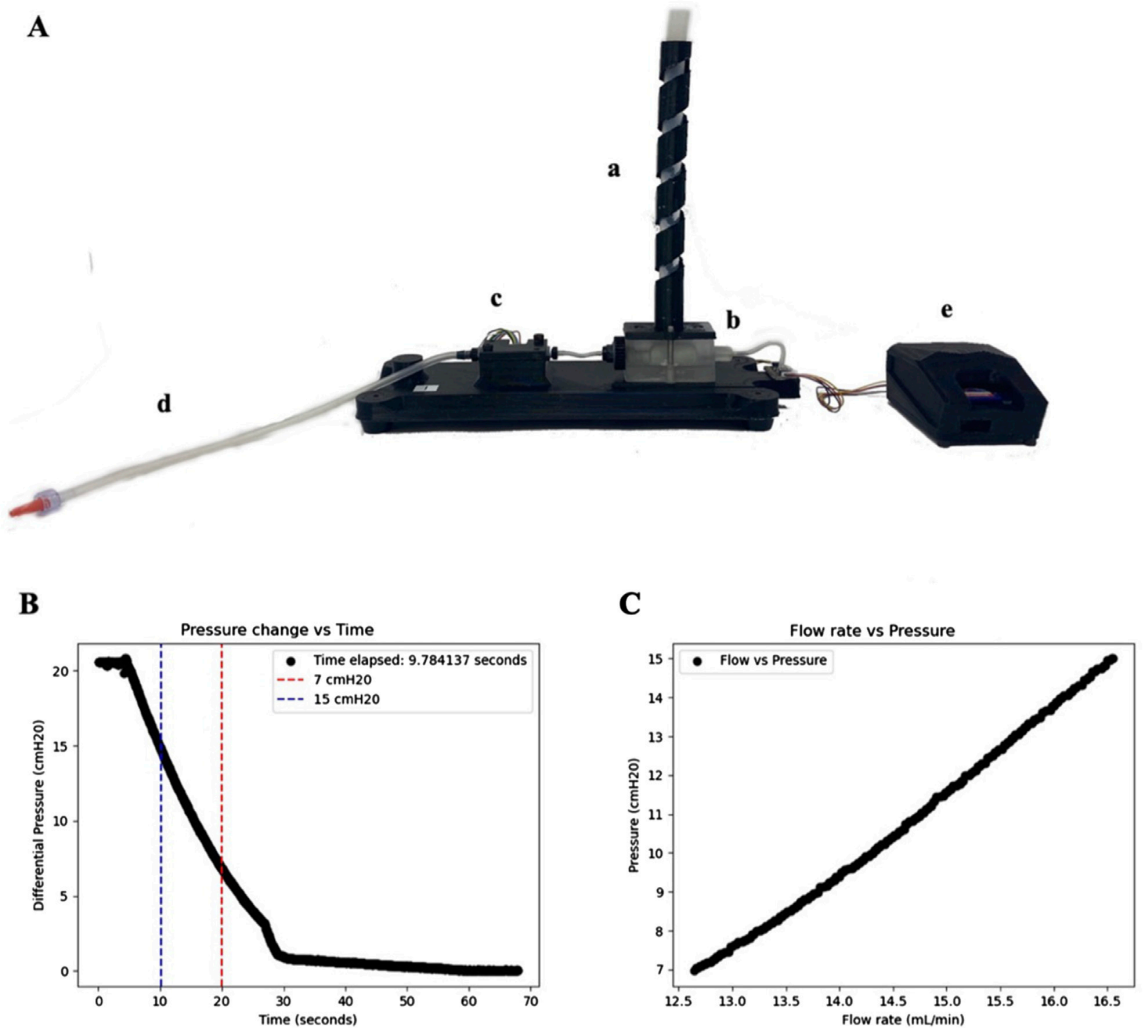
Ventricular Catheter Testing Device (VCTD). **(A)** The major parts of the VCTD setup include the water column (a), a chamber that contains sample catheters (b), a flow sensor chamber (c), a fluid drainage tube (d), and an Arduino board (e). **(B)** Illustration of a pressure vs. time graph generated from experimental data using a standard (S) catheter. The data within the region demarcated by the red and blue vertical lines was selected for analysis, representing the time interval during which the hydrostatic pressure decreased from 15 hPa to 7 hPa. **(C)** Pressure vs. flow graph constructed from the same catheter data. A rolling average of 110 data points was applied to the flow data to mitigate noise and improve measurement accuracy. This approach was implemented because the flow sensor collects more data points than required, and the rolling average effectively filters out noise while preserving the integrity of the measurements.

Catheters were tested using the Ventricular Catheter Testing Device (VCTD), a gravity-driven, portable benchtop model that utilizes a fixed hydrostatic pressure range to quantitatively analyze relative resistance to fluid flow between catheter designs (Figure 5) (Gopalakrishnan et al., 2023). The VCTD collects data on pressure, flow rate, and time. While the VCTD provides valuable insights, it is important to note that the VCTD cannot replicate the exact resistance a catheter would encounter within the ventricles of varying size, as it lacks the variable properties inherent to the ventricular environment. As such, the resistance observed from a fixed pressure drop in the VCTD—referred to as ‘relative resistance'—is used as a proxy measurement rather than an absolute measure of the catheter’s resistance *in-vivo* (Gopalakrishnan et al., 2023). We report these shifts in relative resistance in terms of time elapsed (s). Specifically, the time elapsed for a set volume of fluid (10 mL) to travel through a set length and tubing diameter (Figure 5A, d) (30 cm length, 5 mm diameter). By controlling the fluid volume, tubing dimensions, and pressure conditions, we were able to systematically compare the resistance of different catheter designs in a consistent manner, isolating the variations in catheter resistance that are attributable to design rather than uncontrolled testing factors. This standardization simplifies the data for clinicians by eliminating other variables, allowing them to focus solely on performance differences such as resistance and flow efficiency. The resulting data more accurately reflects the relative resistance catheters might encounter in clinical settings, making it easier for clinicians to interpret and facilitating more informed decision-making in selecting catheters for patient care (Gopalakrishnan et al., 2023).17 variations of catheters were tested, with five individual and new catheters tested for each variation (n = 5). Each catheter was tested three times, with the results averaged (totaling 15 trial runs for testing potential intra- and inter-variability). One control group of unused, unexpired catheters 23-cm, barium-impregnated standard ventricular catheters was also tested to provide a commercial reference standard against the catheters specifically manufactured for this study.

Using methods from our previous study for data collection, we analyzed time-elapsed data by plotting pressure (hPa) against time (seconds) (Gopalakrishnan et al., 2023). Data collection began when the hydrostatic pressure started to decrease from 15 hPa and ended when it reached 7 hPa. This allowed us to measure the time required for the pressure drop, which was used as a metric for “relative resistance” (Figure 5B). To calculate catheter resistance, pressure was plotted against flow rate (μL/min), and the slope of the linear region was used to determine the resistance (R) (Figure 5C). This resistance was then converted from hPamin/μL to Pas/m³ using dimensional analysis, ensuring consistency with units used in the literature. The calculated resistance values were validated by comparing them to known literature values (Gopalakrishnan et al., 2023).

### Blood-induced blockage of catheters

**FIGURE 6:**
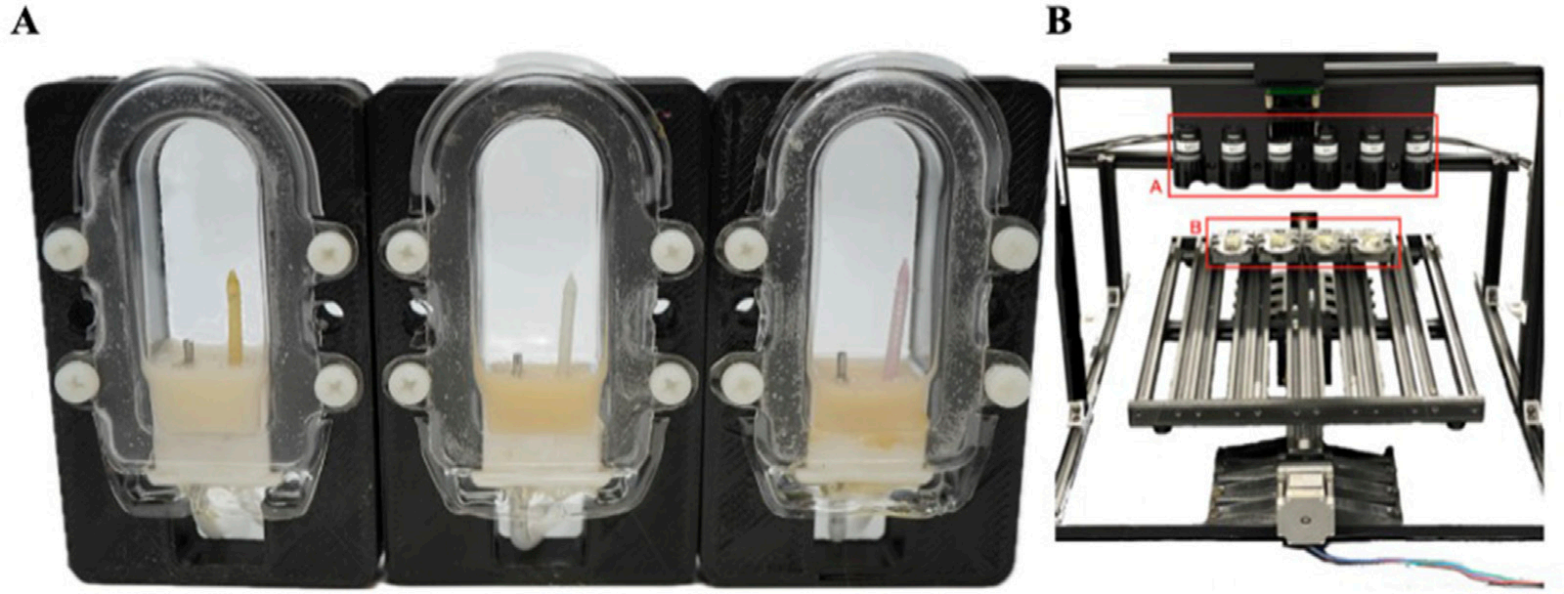
Blood assay chambers and Catheter Obstruction Monitoring Device (COMD). **(A)** Chambers contained catheters with varying lumen diameters: 0.86mm (LD1) on the left, 1.36mm (S) in the center, and 1.86 mm(LD2) on the right. **(B)** Major components of the setup include the microscope camera (A), and the chambers positioned directly under the microscope cameras, spaced 15 cm apart (B).

To emphasize the importance of the architectural influence of catheters, LD1, S, and LD2 were selected for blood-based biologic assessment due to its wide range of relative resistance observed (Table 2). These catheters were placed in Polyethylene Terephthalate Glycol (PETG) chambers (Figure 6A) according to previously reported methods. The chambers were set to the Catheter Obstruction Monitoring Device (COMD) (Figure 6B), where the obstruction was recorded and rendered into quantitative data. A 2.5% blood concentration was employed in this experiment to accelerate the observation of flow obstructions across catheter variants with differing lumen diameters.

**TABLE 2:** Time elapsed data and resistance calculations for all variants presented in the study (with standard deviations). No flow was detected for CO as it was completely obstructed.

Identifiers	N	Mean time elapsed ± standard deviation (s)	Time elapsed range (s)	Relative resistance ± standard deviation (Pa·s/m^3^, 10^10^)
S	5	9.16 ± 0.44	1.07	1.21 ± 0.21
1RO	5	9.29 ± 0.46	1.11	1.24 ± 0.08
2RO	5	9.09 ± 0.38	1.01	1.68 ± 0.28
3RO	5	10.46 ± 0.63	1.67	1.81 ± 0.12
CO	3	−	−	−
Sg.1	5	8.75 ± 0.31	0.71	1.63 ± 0.17
Sg.2	5	9.01 ± 0.25	0.61	1.48 ± 0.20
Sg.3	5	8.77 ± 0.26	0.54	1.64 ± 0.42
Sg.4	5	8.67 ± 0.17	0.46	1.48 ± 0.12
HD1	5	10.91 ± 1.79	4.48	1.79 ± 0.18
HD2	5	8.51 ± 0.11	0.29	1.43 ± 0.25
HD3	5	8.34 ± 0.20	0.41	0.77 ± 0.14
LD1	5	16.90 ± 3.13	8.19	1.26 ± 0.17
LD2	5	7.83 ± 0.17	0.43	0.80 ± 0.04
LO1	5	14.40 ± 4.17	9.14	1.37 ± 0.26
LO2	5	12.74 ± 0.83	2.16	1.29 ± 0.07
LO3	5	80.03 ± 35.32	94.58	4.76 ± 1.57

The experimental system operated at room temperature (25°C) due to its benchtop model constraints. Saline was prepared by dissolving 9 g of NaCl in 1 L of DI water. Two solutions were prepared: one containing blood mixed with saline at twice the required concentration, and another with a coagulant mixture of thrombin, protamine sulfate, and saline. These solutions were connected to the chamber inlet through a four-way connector, and the system was primed for 5–10 min before testing to ensure stability. Shunt failure criteria were set at a threshold of 30 cmH2O. Flow and pressure data for each sample were monitored and recorded in real time using a Sensirion USB Sensor Viewer (Sensirion, Switzerland) and HD700 Data Acquisition software (Teledyne FLIR LLC, United States). The duration from initial exposure to the blood-saline solution until reaching the 30 cmH2O threshold was documented as the time required for full obstruction. Obstruction formation was also observed in real time using the COMD setup.

### Statistical analysis

Data compilation was performed using Microsoft Excel for Windows, while statistical analyses were conducted with SPSS version 28 for Windows, applying a significance level of α = 0.05. To assess the normality of the time-elapsed data, both histogram analyses and formal tests, including the Kolmogorov–Smirnov and Shapiro–Wilk tests, were conducted. Homoscedasticity was confirmed using the Levene Test of Homogeneity of Variances. Data sets that satisfied both normality and homoscedasticity assumptions were analyzed using parametric methods, while data sets that did not meet these criteria were analyzed using non-parametric methods.

An Independent Samples t-test was employed for parametric data to compare the means between two independent groups. This test was used only when the analysis involved two groups and when the data satisfied the assumptions of normality and homogeneity of variances. A one-way ANOVA was employed for parametric data to identify significant differences among experimental groups, followed by a Tukey *post hoc* test for pairwise comparisons where applicable. For data that did not meet normality or homoscedasticity requirements, non-parametric tests such as the Mann-Whitney U test and the Kruskal–Wallis test with pairwise multiple comparisons were applied.

To further explore relationships, a bivariate correlation matrix was generated, screening for significant two-tailed Spearman’s Rank Correlations between variables and the measured time elapsed. Robust regression models were then used to examine the association between ventricular catheter architecture and relative resistance. Data distributions were visualized using box-and-whisker plots generated in SPSS.

## Results

### Commercial and manufactured standard catheter comparison

**FIGURE 7:**
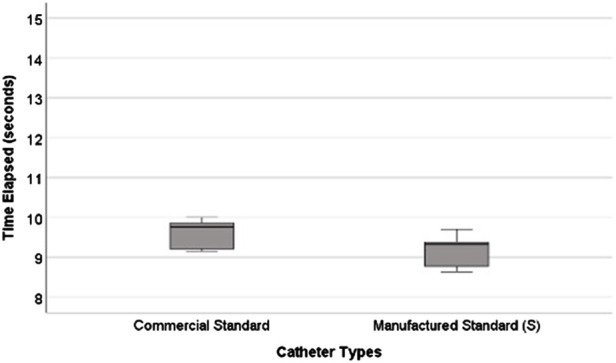
Commercial and manufactured standard catheter time elapsed. The commercial catheter on the left side had an average time elapsed of 9.59 s (Gopalakrishnan et al., 2023). Manufactured catheters were on the right and had a slightly lower average time elapsed 9.17 s (Table 2).

Serving as a surrogate for relative resistance, the mean time elapsed for the manufactured catheter is statistically insignificant (*P* > 0.05) when compared to the commercial catheter’s mean time elapsed (Figure 7) (Gopalakrishnan et al., 2023). Table 2 shows mean time elapsed and mean resistance for all samples (Figure 7).

### Comparison of standard commercially available catheters and various forms of obstructed (deleted) holes: row, complete, and segmental

**FIGURE 8:**
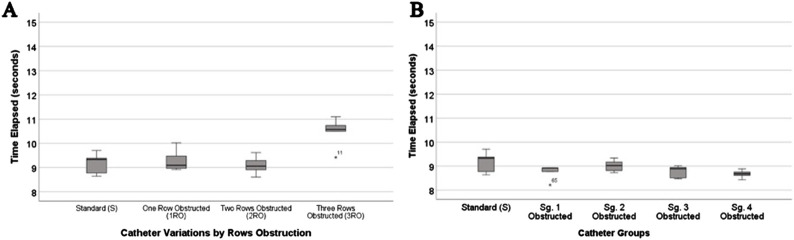
Collage of Row and Segment obstruction experiment results. **(A)** Box-and-whisker plot of the row obstructed the catheter’s time elapsed. From standard, 1 row obstructed, 2 rows obstructed, and 3 rows obstructed, to complete obstruction of all rows (not shown, since no flow was passed through the catheter). **(B)** Box-and-whisker plot of segment obstructed catheters in comparison to standard catheter’s time elapsed.

The *post hoc* analysis (Figure 8A) revealed a significant increase in the mean time elapsed for the 3RO compared to all other RO variations and the standard (*P* < 0.0001). In three samples (n = 3), full obstruction (CO), resulting in no recorded flow within 10 min. These fully obstructed samples were excluded from the statistical analysis. However, it is reasonable to assume that its time elapsed is significantly higher than the standard and all RO variations, consistent with findings from prior studies (Gopalakrishnan et al., 2023). Moreover, the time elapsed data for all variations with hole segment obstructions (Sg.1, Sg.2, Sg.3, Sg.4) and the standard did not exhibit significant differences from each other (refer to Figure 8B).

### Effects of hole and lumen diameter on resistance to flow

**FIGURE 9:**
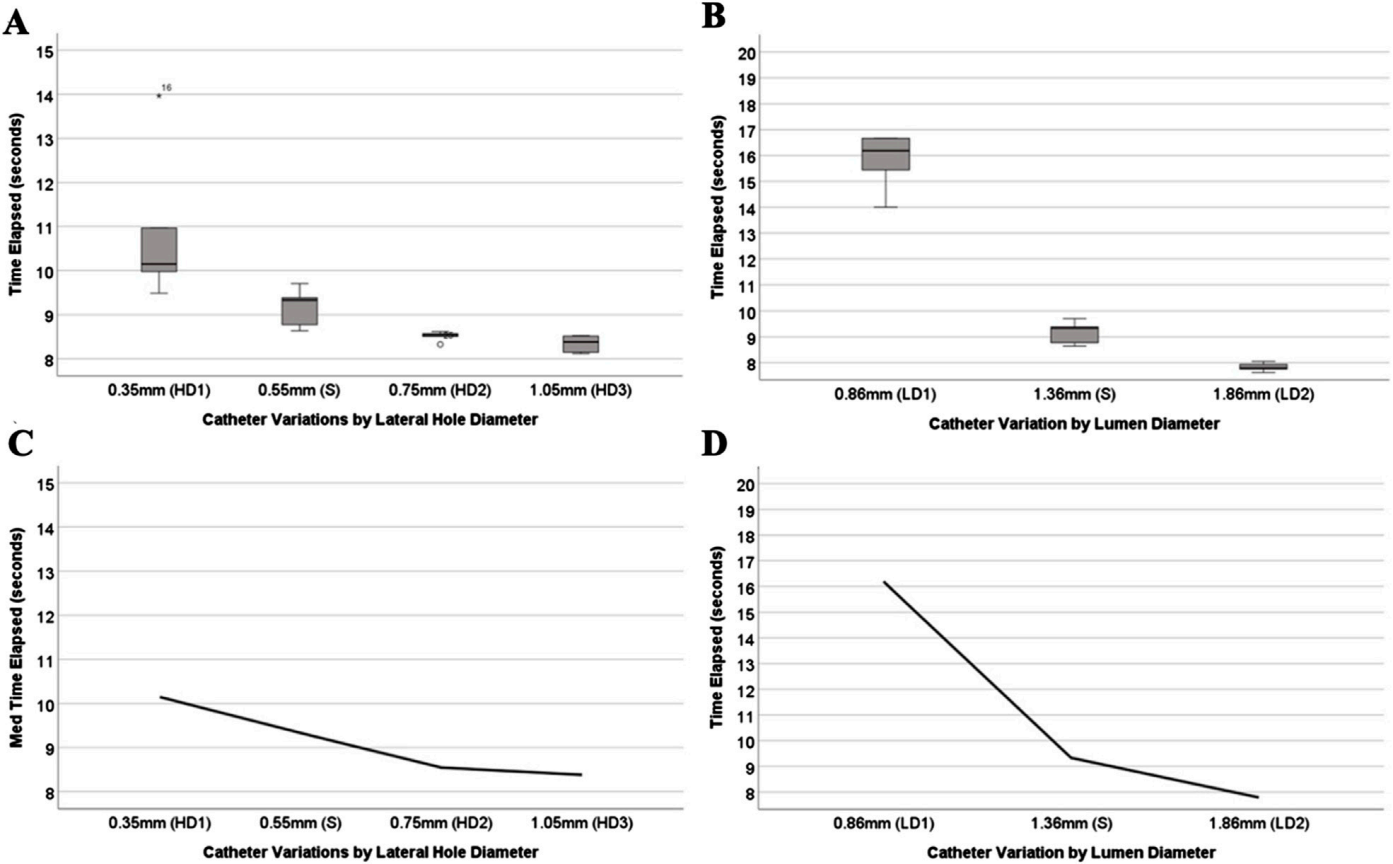
Collage of hole and lumen diameter variation experimental results. **(A)** Box-and-whisker plot of the mean time elapsed for catheters varying in hole diameter. **(B)** Box-and-whisker plot of the mean time elapsed for catheters varying in lumen diameter. **(C)** The trend in relative resistance between each hole diameter variation. **(D)** The trend in relative resistance between each lumen diameter variation.

The experiments investigating manipulation of hole diameter (Figure 9A) revealed significant discrepancies in the time-elapsed data across various hole diameter variations (HD1, S, HD2, and HD3, *P* < 0.001). Notably, as the hole diameter increased, the time elapsed exhibited an exponential decrease. Similarly, the experiments focusing on altering the inner lumen diameter (Figure 9B) displayed significant differences in time-elapsed data among different inner lumen diameter variations (LD1, S, and LD2) (*P* < 0.002). It was observed that with an increase in the inner lumen diameter, the time elapsed decreased exponentially.

### Impact of luminal obstruction on relative resistance

**FIGURE 10:**
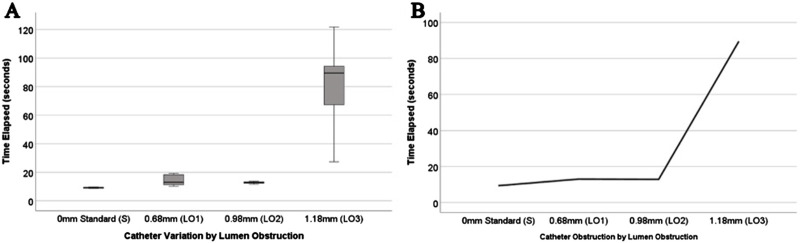
Experimental results of mean time-elapsed for lumen obstruction group. **(A)** Box-and-whisker plot of the mean time elapsed of catheters that have varying sizes of lumen obstructions compared to the standard. **(B)** The trends in relative resistance as the lumen obstruction increases in diameter.

The results shown in Table 2 show that each LO variation’s time-elapsed variances were statistically significant (*P* = 0.001). Pairwise comparison analysis (Figure 10) shows that the meantime elapsed for LO3 is significantly higher from the standard catheter (S) (*P* < 0.05). However, LO3 was not significant to both LO1 and LO2 (*P* > 0.05). Furthermore LO1, LO2, and the standard (S) was not significant to each other (*P* > 0.05).

### The impact of architectural manipulations on time-elapsed

**FIGURE 11:**
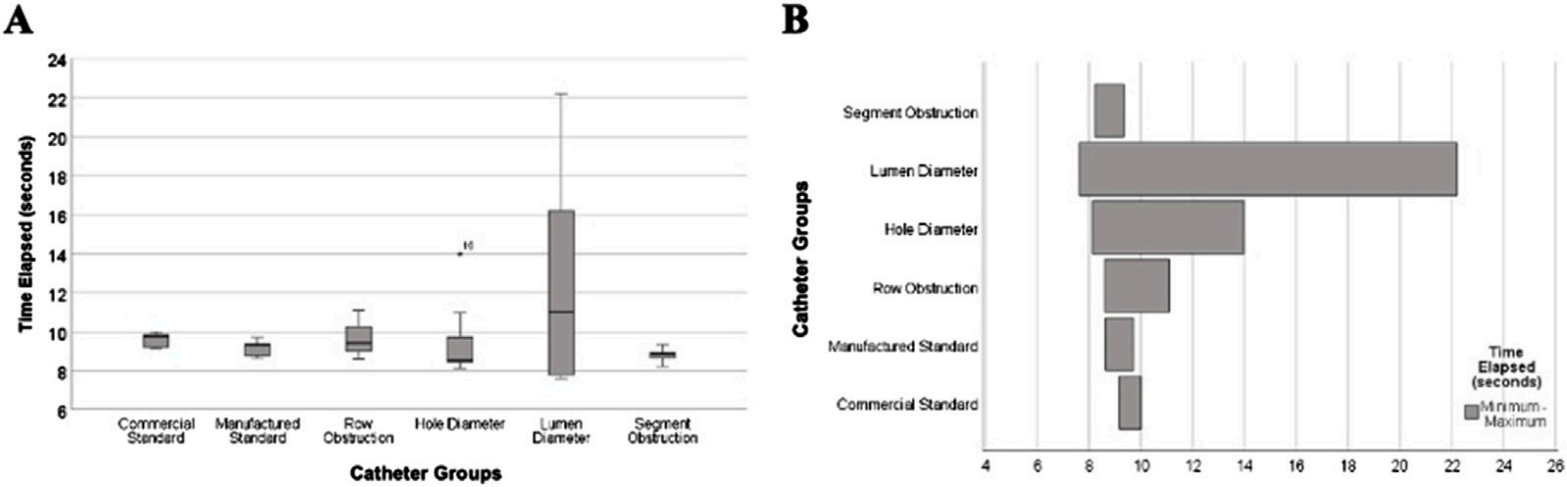
Time elapsed ranges from all catheter groups with altered architecture and control. **(A)** Box-and-whisker plot showing the ranges in mean time elapsed from the Ventricular Catheter Testing Device (VCTD) for obstruction in commercial control, manufactured control, and other catheter groups with varying architectures. **(B)** Horizontal bar graph illustrating the range of time elapsed (minimum to maximum) for each catheter group. Variations in time are influenced by differences in lumen diameter, hole diameter, and obstruction types.

The results demonstrate significant differences in the ranges of time elapsed between groups (*P* < 0.034). Notably, catheters with lumen diameter modifications exhibited the widest range, spanning from approximately 8 s to over 22 s. Hole diameter modifications showed a moderate range of 8–14 s, reflecting a middle ground compared to other groups. In contrast, catheters with segment obstruction showed consistently low ranges of 7–9 s. Commercial standard catheters and manufactured standard catheters demonstrated narrow and comparable ranges of 9–11 s (Figures 11).

Bivariate correlation matrix revealed a strong negative correlation between inner lumen diameter and time elapsed (Spearman’s ρ = −0.627, *P* < 0.001), as well as between hole diameter and time elapsed (Spearman’s ρ = −0.533, *P* < 0.001). The number of rows of holes exhibited a weaker negative correlation with time elapsed (Spearman’s ρ = −0.352, *P* = 0.004). Robust regression analysis identified significant relationships between catheter architectural variables and time elapsed within the resistance setup (n = 45). Inner lumen diameter showed the largest effect, with a coefficient of −2.162 (*P* < 0.001), indicating a significant reduction in time elapsed with larger inner diameters. Hole diameter demonstrated a coefficient of −1.454 (*P* < 0.001), indicating a notable reduction in time elapsed. The number of rows of holes had a smaller but significant effect, with a coefficient of −0.385 (*P* < 0.001).

### Impact of inner lumen diameter on time to hemorrhagic obstruction

**FIGURE 12:**
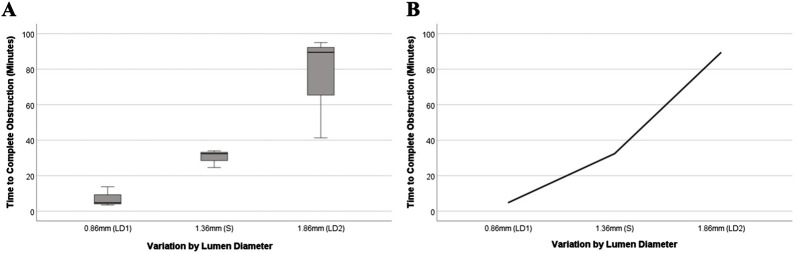
Blood assay on varying lumen diameter catheters. **(A)** Box-and-whisker plot showing the mean time until complete flow obstruction for catheters with varying lumen diameters compared to the standard. **(B)** The trends in relative time till complete obstruction of the catheter.

The results shown in Figure 12 indicate that catheters with varying inner lumen diameters have statistically significant differences in time elapsed till complete obstruction to flow (*p* = 0.027). The results indicate that for the 0.86 mm (LD1) group, the mean time to complete obstruction was 7.33 ± 5.59 min. In the 1.36 mm (S) group, the mean time was 30.33 ± 5.04 min. Lastly, for the 1.86 mm (LD2) group, the mean time to complete obstruction was 75.27 ± 29.53 min.

## Discussion

The effects of catheter geometry on fluid flow dynamics have been described in several studies, some of which even offer potential alternate designs. The literature strongly suggests that the current design specifications of ventricular catheters leave much room for improvement and may even play a significant role in catheter failure. Lumen diameter, hole number, hole diameter, and hole configuration are most often highlighted in the literature as contributing to increased resistance and/or increased obstruction (Thomale et al., 2010; Galarza et al., 2018; Harris and McAllister, 2011). Adjustments in these parameters are very likely to improve the performance of ventricular catheters. As hydrocephalus treatment moves forward, an evidence-based data-driven approach to catheter design is likely to provide a significant benefit to patient outcomes.

In this study, we investigated the relative resistance of fluid outflow from catheters with and without architectural modifications to determine how varying the initial design of the catheter would influence the inherent resistance of the shunt system. All the catheters made in this study were based on commercially available catheters but were then systematically varied by one element at a time (Table 2). We used time elapsed as a proxy for relative resistance, as described in prior work using the VCTD (Gopalakrishnan et al., 2023). Our manufactured standard was not significantly different from the commercial catheter in relative resistance, establishing that the novel Catheter Research and Fabrication Technology System (CRAFTS) produces catheters within the outflow performance range of commercial catheters. This allows us to make inferences about the relationship between commercial catheter geometries and changes in fluid flow using data from the catheters constructed and presented in this study.

### Effects of geometric parameters on relative resistance

Changes in a catheter’s resistance to fluid outflow were insignificant until three rows (3RO) were blocked (Figure 8A). Unpublished computational fluid dynamics results from our lab indicate a significant increase in resistance after 90% of the holes are occluded. Although the resistance became too high to measure in fully occluded samples, the lack of statistical significance from 2RO to 3RO may be because the highest occlusion achieved was only 75%, falling short of complete blockage. Hole diameter also showed marked decreases in relative resistance as the diameter increased (Figure 9A). Among the manipulated parameters, those that had the greatest impact on relative resistance were lumen diameter and lumen obstruction (Figures 9B, 10A). Most notably, the results of all these experiments show nonlinear changes in relative resistance, even when parameters are changed linearly (e.g., lumen diameter). These data are consistent with our previous work which observed that the effect of obstruction on relative resistance to fluid flow is nonlinear (Gopalakrishnan et al., 2023). A study from Lee et al. also found results that suggested a drastic increase in resistance when the catheter nears 100% occlusion (Lee et al., 2022).

From a clinical perspective, catheters with at least one unobstructed row can maintain adequate flow and not add to inherent resistance of the shunt system. However, flow and pressure gradients across catheter holes and within the catheter lumen will shift over time with changes to the degree of partial or full occlusion. We are actively studying how resistance is shifted from explanted patient catheters. Finally, we must also consider that downstream effects caused by transient changes in the number or degree of patency in each hole or through the lumen are not yet fully understood. Our upcoming work in this area will evaluate the impact that varying resistance, flow patterns, pressure gradients, and shear may have on all protein adsorption isotherms and attached or actively attaching cells.

### The impact of architectural manipulations on time-elapsed

The observed ranges in time elapsed across catheter groups emphasize the significant influence of specific architectural modifications on resistance within the setup. As shown in Figure 11, lumen diameter alterations demonstrated the broadest range in time elapsed, indicating its substantial role in modulating resistance. Hole diameter and number of rows, while also impactful, displayed more moderate ranges, suggesting a less pronounced effect on lumen relative resistance. These ranges provide valuable insight into the hierarchy of variables affecting resistance. If optimizing catheter performance is the goal, prioritizing modifications to lumen diameter is paramount, as the data clearly show this variable has the most significant influence on resistance time (Yang Y. et al., 2022). Variance in hole diameter will impact other parameters outside the scope of this work and should continue to be explored.

The relevance of these findings is further supported by the robust regression analysis, which highlights lumen diameter as the most impactful variable to relative resistance. Beyond its broad range in time elapsed, lumen diameter also exhibited the largest regression coefficient, confirming its strong association with resistance (Galarza et al., 2013). Hole diameter and the number of rows of holes, while statistically significant, had smaller coefficients, indicating a less substantial role in reducing relative resistance. These findings demonstrate that modifying lumen diameter is not only critical for creating variability in resistance but also for achieving the most pronounced reductions in resistance within the setup (Yang Y. et al., 2022).

The importance of these observations is magnified when considering the cross-sectional area of the catheter relative to the total flow area as shown in Figure 4. For the standard catheter (S), 70% of the cross-sectional area is occupied by the catheter material, leaving only 30% available for flow. Similarly, the smallest lumen diameter catheter (LD1) has 88% of its cross-sectional area taken up by the catheter material, leaving just 22% for flow. In contrast, the largest lumen diameter catheter (LD2) occupies only 42% of the cross-sectional area with material, allowing 58% for flow. These measurements were calculated using their respective CAD (Computer-Aided Design) files, providing precise and accurate estimates of the cross-sectional proportions. Larger inner lumen diameters result in a lower cross-sectional area percentage, thereby decreasing resistance within the system. This relationship provides mechanistic evidence supporting the superiority of larger lumen diameters in improving catheter performance. By minimizing the proportion of the catheter obstructing the flow path, time-elapsed is reduced, facilitating better fluid dynamics (Galarza et al., 2015). These findings underscore the practical implications for catheter design: optimizing lumen diameter and associated features such as hole diameter can significantly enhance performance, offering a targeted approach to improving catheter efficacy (Yang Y. et al., 2022).

### Practical value of decreased catheter resistance

Several previous studies have described increases in ventricular catheter resistance to fluid flow over time (Cheatle et al., 2012; Gopalakrishnan et al., 2023; Galarza et al., 2018). Further research is needed to fully understand the clinical significance of the ventricular catheter’s role on resistance. For example: is increased resistance directly contributing to catheter obstruction, or is increased resistance a marker of increasing obstruction? The current literature suggests both hypotheses are true, and while an exact quantitative relationship between resistance and catheter performance remains to be found, the literature does report that cellular adhesion is flow-dependent and both increased and decreased shear stress (to a threshold) can promote astrocyte attachment (Harris and McAllister, 2011; Lee et al., 2020). Additionally, proximal ventricular catheters have been shown to become more resistant to fluid flow over time, even when not exposed to CSF (Cheatle et al., 2012). The literature on mechanisms explaining this phenomenon is sparse; there is a lack of practical methods to longitudinally and quantitatively compare ventricular flow through *in situ* catheters to sterile, unused catheters. Further experimentation involving longitudinal comparisons of unused shunts and shunts exposed to CSF (i.e., explanted shunts) would yield great insight into potential catheter “aging.” There is likely great practical value in modulating resistance to reduce cell adhesion and maintain CSF outflow over longer periods.

When discussing fluid flow through ventricular catheters, there is often a concern about over-drainage. Indeed, over-drainage is associated with its own negative acute and chronic symptoms (Ros et al., 2021). Preventing over-drainage is the main function of the valves and gravitational units used as part of shunt systems. Combining current *in-vitro* models of catheter fluid flow with models of varying valve function is likely to lead to reduced rates of both shunt failure and shunt over-drainage. The literature already contains descriptions of modular testing chambers that can accommodate such experimental designs, including experiments involving blood or human cell cultures (Menkara et al., 2022). Integrating data-driven catheter design into a holistic approach that includes valve function analysis in models that better simulate *in-vivo* CSF flow should yield very promising insights and is an area of upcoming research.

### Blood assay: ventricular catheter design & hemorrhage

The blood assay results indicate that increasing the lumen diameter from LD1 to LD2 (while maintaining the same outer diameter) led to an average tenfold increase in the time required for the catheter to become completely obstructed (Figure 12). This finding aligns with the results from the resistance setup, which demonstrated that as the luminal diameter increased, the resistance to flow through the catheter decreased significantly (Figure 9B). The blood assay serves as a supplementary evaluation method to assess the durability and longevity of a catheter.

External ventricular drainage (EVD) is a mainstay for treating the many causes of increased ICP, including hydrocephalus. Infection and hemorrhage are the most common complications of EVD. Previous research suggests a similar effect with isolated changes of external diameter. For instance, Woo et al. noted the paucity of literature detailing risk factors for EVD-related hemorrhage and performed a multicenter study with EVD-related hemorrhage as the endpoint of inquiry. Most interestingly, their data indicated that the only two independent surgical-sided predictors of EVD-related hemorrhage were catheterization accuracy and catheter design (Woo et al., 2019). Among the three brands of catheter design Woo et al. tested (from Codman, Integra, and Medtronic), the catheter with the smallest outer diameter was found to have the least risk of hemorrhage (Woo et al., 2019). One of the main advantages of our approach was that the outer diameter of the LD1 and LD2 catheters remained the same as that of the S catheters, allowing the larger lumen to be achieved without increasing the outer diameter. This design, therefore, did not introduce a potential risk of hemorrhage due to an increased outer diameter.

Exposure of ventricular catheters to blood is practically unavoidable, whether from mechanical injury caused by complications of hydrocephalus or from surgical implantation of a device into a highly vascular organ. The presence of blood in fluid flow through a ventricular catheter changes the flow profile through the catheter hole interfaces and the lumen. Additionally, adsorption of proteins and other blood components over time will contribute to changes in shear stress (Harris and McAllister, 2012). Blood clots are one of the known contributors to catheter obstruction, and the addition of CSF to blood makes blood hypercoagulable. Vandersteene et al.’s work indicated that a 5%–9% concentration of CSF is sufficient to cause a significant increase in coagulability (Vandersteene et al., 2019). Increased shear stress (e.g., from turbulent flow through a stenotic catheter) is also known to increase thrombogenesis in catheters exposed to blood (Lucas et al., 2014; Clark et al., 2015). It should be noted that larger hole diameters may increase the risk of whole tissue obstruction entering via the holes and into the catheter lumen, but this direct cause-effect has not been exhaustively investigated. A computational fluid dynamics assessment is necessary to understand if and how hole diameter will cause shifts in pressure gradients and tissue deformation in addition to changes in resistance. This is also likely a function of the number of holes and the proximity of the holes to the tissue interface itself and is an ongoing area of investigation by our group. Considering that our results and those of Woo et al. suggests that changes in inner and outer diameters have tangible clinical ramifications regarding fluid outflow through a catheter, there is likely a way to optimize inner diameter, outer diameter, and hole interface configuration to reduce flow-related shear stress and subsequently reduce the risk of blood clotting. These optimizations may also be able to reduce the risk of EVD-related hemorrhage and luminal obstruction caused by matter entering through the hole interfaces.

The 2.5% blood concentration used in this study, though not physiologically relevant, it was selected to expedite results (Yang Q. et al., 2022; Devathasan et al., 2021). A previous clinical study reported a range of 25–250 red blood cells/μL in CSF, this roughly translates to 0.05%–0.5% concentration (Fam et al., 2017). Additionally, the experimental setup operated at room temperature (25°C), constrained by the benchtop model. This non-physiological temperature may have influenced the interaction between blood, saline, and thrombin, potentially altering clotting dynamics and reaction rates. Conducting future experiments at body temperature (37°C) would better simulate physiological conditions and provide results more reflective of *in vivo* behavior. The focus of the experiment was to assess relative obstructions among catheter designs, rather than replicating physiological resistance to flow. Additionally, the blood assay served as a secondary screening method following VCTD resistance results to further evaluate how catheter architecture influences its functionality and resistance. Future research should model the effects of varying blood concentrations on coagulability, shear stress, and catheter obstruction, combining this with physiological temperatures.

### Standardized placement and simplification of luminal obstructions

The ball-point obstruction in the catheter luminal obstruction groups was strategically placed following the last segment of holes furthest from the catheter tip, as illustrated in Figure 2C, where the location is marked by a red circle. This placement was chosen for simplicity and to provide preliminary results while avoiding the complexities of more variable obstruction scenarios, such as obstructions within random holes, varying degrees of hole obstruction, or irregular positioning along the lumen. While this approach introduces a limitation in fully replicating the complexity of realistic occlusions, it allowed for controlled experimentation to investigate the relationship between obstruction and resistance.

For this study, inner lumen obstructions were simplified to a ball-point design to standardize the introduction of resistance, which could then be correlated with the cross-sectional area of the obstruction (Figure 12). Future studies should aim to incorporate more intricate and physiologically realistic occlusion models, such as partial obstructions within individual holes or irregularly shaped obstructions, to provide a deeper understanding of the effects of luminal obstructions on resistance and flow dynamics. These enhancements would enable a more comprehensive analysis of how complex obstructions influence catheter performance under clinically relevant conditions.

### Catheter production process limitations

Novel, in-house, small scale catheter production faces many difficulties. Although CAD provides considerable design flexibility, there are technical difficulties at subsequent stages. Precision is impacted by 3D printing resolution, which might cause variations from CAD models. Resin shell cleaning can be challenging, particularly for designs with smaller openings. Plaster casting may create air pockets, which increases the possibility of lumen collapse during firing. The extraction of silicone causes tiny air bubbles to form, which might damage the integrity of the catheter’s structure. When cleaning catheters in a manual process, delicate silicone walls could be harmed.

It is well known that surface roughness or irregularities can manipulate fluid flow in turbulent flow conditions (Liu et al., 2019; Gloss and Herwig, 2010; Zhou and Yao, 2011). While some degree of surface roughness is present on manufactured catheters, fluid flow through the ventricles has cardiac driven pulsatility with laminar flow (Reynolds number less than ten) (Li, 1988). Still, future work continues to reduce any manufactured catheter irregularities. To improve catheter quality and performance, continuous process optimization is required. Although the specified dimensions in CAD models did not perfectly translate into physical catheters due to 3D printing limitations, the overall trends from CAD designs were maintained. While absolute precision may vary, relative differences between design variants were preserved, allowing for reliable comparative analysis of catheter performance. Future research may benefit from advancements in production technologies to improve accuracy.

The obstructions built into the manufactured catheters are rigid bodies, whereas biological tissue exhibits a range of porosities and extracellular matrix densities that can affect the flow of CSF around or into a lesion. These rigid bodies serve as a proof of concept to investigate variables influencing resistance to CSF flow. The blood assay experiments further provide preliminary results using a better approximation of *in-vivo* conditions. Future work will test the relative resistances of failed catheters exhibiting different obstruction patterns. Another limitation is the use of silicone for the obstructive masses, which does not replicate the density or viscoelastic properties of biological tissue. Future studies should use tissue-specific models, as previous research shows that obstructing tissue is dense with little extracellular matrix (Barthold et al., 2021).

### Potential impact of silicone pigment dye on blood assay accuracy

A potential limitation of our study is the unknown impact of silicone pigment dye within the catheter on blood flow dynamics and assay accuracy. While no visible leaching was observed during the experiment, we have not conducted specific tests to verify whether the dye might interact with blood components or influence assay results over time. Future studies could include spectrophotometric analysis and hemoglobin level monitoring to ensure that the silicone pigment remains stable and does not leach into the blood under the tested conditions (Martinek, 1965; Iwamaru et al., 2001; Ihnat, 1982). These additional tests would help confirm that the pigment does not interfere with the accuracy of the assay.

### Alternative catheter production methods

Catheter production has been dominated by hole-punch fabrication methods involving the use of blunt-ended needles to puncture holes in PDMS tubing (Harris and McAllister, 2011; Lintula, 2008; Medtronic Inc, 2006). This method created innate rough surfaces amongst holes that potentially aid in the adhesion of macrophages and astrocytes (Harris and McAllister, 2011). External modifications of PDMS through electrospun polyurethane (EPU) present an alternative to conventional PDMS catheters, but the reduction in hydraulic permeability due to potential cell growth poses a significant challenge for this technology (Suresh and Black, 2015). Although the use of epoxy resin or hot glue may permit occlusion of holes to test for relative resistance to flow, the same catheters under biological conditions norm to hydrocephalus may influence the reactivity of various obstruction-causing facets, which may confound results (Lee et al., 2022; Gopalakrishnan et al., 2023). One notable and innovative method of producing catheters involves the use of two-photon lithography (TPL) for three-dimensional fabrication in nanoscale resolution (Harinarayana and Shin, 2021). However, the innate nature of a catheter to be thin and flexible with small features (holes) can be more challenging to fabricate when printing in high resolutions (Bunea et al., 2021).

Our CRAFTS addressed many of these limitations by having the ability to create catheters with virtually any design and biocompatible material (including low-durometer materials). Although in this study 50D silicone was the only material used, future studies will use different biocompatible materials to produce catheters that can be tested for relative resistance and compatibility in blood assays. Furthermore, the high-throughput CRAFTS will be established, and using parametric data from this study optimal catheter will be designed.

## Conclusion

In this study, the dynamic versatility of a novel catheter manufacturing process was employed to systematically investigate the impact of architectural features of catheters. A blood assay was conducted on the catheters varying in lumen diameter to simulating artificial hemorrhagic hydrocephalus. The experiments revealed a significant impact of hole and lumen diameter variations on relative resistance, with inner luminal obstructions also strongly influencing resistance to flow. These effects followed a non-linear trend. While row obstructions caused slight changes in resistance, variations in the segmental hole were not significant. Blood assay results further demonstrated a significant difference in the time to complete obstruction based on inner lumen diameter. Future studies will concentrate on refining CRAFTS and focus on optimizing catheter design based on these parametric results. Furthermore, we will explore different materials to assess their impact on catheter resistance and efficacy in biological environments.

## Data Availability

The original contributions presented in the study are included in the article/Supplementary Material, further inquiries can be directed to the corresponding author.
