# Correction: The impact of architectural modifications on relative resistance to fluid flow in ventricular catheters

**DOI:** 10.3389/fbioe.2026.1769505

**Published:** 2026-01-12

**Authors:** Rajesh Kumar Madhavan, Ahmad Faryami, Nathan Tappen, Pranav Gopalakrishnan, Shaheer H. Ajaz, Christopher Roberts, Carolyn Harris

**Affiliations:** 1 Department of Biological Sciences, Wayne State University, Detroit, MI, United States; 2 Department of Biomedical Engineering, Wayne State University, Detroit, MI, United States; 3 Department of Chemical Engineering, Wayne State University, Detroit, MI, United States; 4 School of Medicine, Wayne State University, Detroit, MI, United States

**Keywords:** ventricular catheter (VC), catheter resistance, bench top model, catheter architecture and design, hydrocephalus

Author Christopher Roberts was omitted as an author in the published article. The correct author list reads:

Rajesh Kumar Madhavan^1^, Ahmad Faryami^2^, Nathan Tappen^3^, Pranav Gopalakrishnan^4^, Shaheer H. Ajaz^2^, Christopher Roberts^3^, Carolyn Harris^3,^*

The Author Contributions Statement has been corrected to read:

RM: Conceptualization, Data curation, Formal Analysis, Investigation, Methodology, Validation, Visualization, Writing – original draft, Writing – review and editing. AF: Conceptualization, Data curation, Formal Analysis, Methodology, Project administration, Resources, Supervision, Writing – review and editing. NT: Conceptualization, Investigation, Methodology, Writing – review and editing. PG: Writing–original draft, Writing – review and editing. SA: Data curation, Visualization, Writing – review and editing. CR: Data Curation, Formal Analysis, Software, Visualization, Writing – review and editing. CH: Funding acquisition, Project administration, Resources, Supervision, Writing – review and editing.

The original article has been updated.

