# Supplementary material for: The impact of architectural modifications on relative resistance to fluid flow in ventricular catheters
Source: Front Bioeng Biotechnol. 2025 Jan 29;12:1519499. doi: 10.3389/fbioe.2024.1519499 (PMC11815352; doi:10.3389/fbioe.2024.1519499)
Supplement: Supplementary file 1 [file DataSheet1.pdf]

Supplemental Data

Table 3. Measure of CAD dimensions of catheter variants. The outer lumen diameter of all catheters was 2.45mm. For the LO groups the corresponding cross-sectional areas are 42,60, and 77%.

Identifiers	# of Rows	# of holes per Rows	Inner Lumen Diameter (mm)	Hole Diameter (mm)	Inner Lumen Obstruction Diameter (mm)
**S**	4	8	1.36	0.55	0
**1RO**	3	8	1.36	0.55	0
**2RO**	2	8	1.36	0.55	0
**3RO**	1	8	1.36	0.55	0
**CO**	0	0	1.36	0	0
**Sg.1**	4	6	1.36	0.55	0
**Sg.2**	4	6	1.36	0.55	0
**Sg.3**	4	6	1.36	0.55	0
**Sg.4**	4	6	1.36	0.55	0
**HD1**	4	8	1.36	0.35	0
**HD2**	4	8	1.36	0.75	0
**HD3**	4	8	1.36	1.05	0
**LD1**	4	8	0.86	0.55	0
**LD2**	4	8	1.86	0.55	0
**LO1**	4	8	1.36	0.55	0.68
**LO2**	4	8	1.36	0.55	0.98
**LO3**	4	8	1.36	0.55	1.18
